# Reconciling disparate prevalence rates of PTSD in large samples of US male Vietnam veterans and their controls

**DOI:** 10.1186/1471-244X-6-19

**Published:** 2006-05-02

**Authors:** William W Thompson, Irving I Gottesman, Christine Zalewski

**Affiliations:** 1 Immunization Safety Office, US Centers for Disease Control and Prevention, Atlanta, GA, USA; 2 Departments of Psychiatry and Psychology, University of Minnesota, Minneapolis, MN, USA; 3 Pacific Graduate School of Psychology, Palo Alto, CA, USA

## Abstract

**Background:**

Two large independent studies funded by the US government have assessed the impact of the Vietnam War on the prevalence of PTSD in US veterans. The National Vietnam Veterans Readjustment Study (NVVRS) estimated the current PTSD prevalence to be 15.2% while the Vietnam Experience Study (VES) estimated the prevalence to be 2.2%. We compared alternative criteria for estimating the prevalence of PTSD using the NVVRS and VES public use data sets collected more than 10 years after the United States withdrew troops from Vietnam.

**Methods:**

We applied uniform diagnostic procedures to the male veterans from the NVVRS and VES to estimate PTSD prevalences based on varying criteria including one-month and lifetime prevalence estimates, combat and non-combat prevalence estimates, and prevalence estimates using both single and multiple indicator models.

**Results:**

Using a narrow and specific set of criteria, we derived current prevalence estimates for combat-related PTSD of 2.5% and 2.9% for the VES and the NVVRS, respectively. Using a more broad and sensitive set of criteria, we derived current prevalence estimates for combat-related PTSD of 12.2% and 15.8% for the VES and NVVRS, respectively.

**Conclusion:**

When comparable methods were applied to available data we reconciled disparate results and estimated similar current prevalences for both narrow and broad definitions of combat-related diagnoses of PTSD.

## Background

The systematic examination of combat-related stress disorders has been ongoing since the end of World War I. [1-5] In 1980, Post-Traumatic Stress Disorder (PTSD) first appeared as a formal diagnosis in DSM-III [6-9] and has periodically been the focus of debate regarding appropriate definition, symptom criteria, and etiology. [10-16] The potential traumas associated with the diagnosis of PTSD are heterogeneous and include military battle, violent sexual attacks, and natural disasters. [17-22] Many studies have demonstrated that PTSD symptoms persist over time and can emerge or re-emerge long after the occurrence of the original trauma, given another "trigger". [19,21,23-36] Certain traumas such as wartime combat or incarceration as a prisoner of war typically lead to more pronounced and longer lasting PTSD symptoms. Individual differences in response to severe traumas are well documented, and both environmental and biological factors have been found to moderate the subsequent experience of stress-related symptoms. [37-45] Finally, a recent study has suggested that rates of combat-related PTSD may be inflated because some individuals report experiencing a traumatic combat exposure when in fact there is little objective evidence to support their claims. [46-48]

A number of different instruments have been developed over the last two decades to measure or diagnose PTSD including the Structured Clinical Interview (SCID), [6,49-51] alternative versions of the PTSD module of the Diagnostic Interview Schedule (DIS), [52-59] the Mississippi Scale for Combat-Related PTSD (MISS-PTSD) [60-64], and the PTSD sub-scale of the Minnesota Multiphasic Personality Inventory (MMPI-PTSD) [65-71]. The instruments used for diagnosing PTSD have varying reliabilities. The psychometric properties of the MISS-PTSD have been studied extensively, supporting the reliability and validity of the instrument. [72-74] The psychometric properties of the MMPI-PTSD have been studied less often and the measure has been criticized for being a non-specific measure of response to severe trauma. [67,75]

The two largest and most comprehensive studies to examine the impact of the Vietnam War on Vietnam-veterans were carried out more than a decade after the US pulled military troops out of Vietnam. The National Vietnam Veteran Readjustment Study [19,76-79] assessed a nationally representative sample of male Vietnam Theater-Veterans, male Vietnam Era-Controls, male civilian controls, and female veterans and civilian controls. The Vietnam Experience Study (VES) [21,80,81] randomly sampled male Vietnam Theater-Veteran Army draftees and male Army Vietnam Era-Control draftees. Although both studies were conducted at approximately the same time and examined large representative samples of male veterans, the published studies reported disparate current and lifetime rates of PTSD. Specifically, the NVVRS reported the current prevalence rate of PTSD to be 15.2% [19,76] whereas the VES reported a current prevalence rate of 2.2%. [21] Previous attempts have been made to describe the similarities and differences between the NVVRS and VES methodologies [82] and the authors concluded that the NVVRS sample was the only study appropriate for extrapolating national PTSD prevalence estimates because the VES used an invalid PTSD diagnostic instrument. The present investigation examined the methodologies used by the two studies and provides a reanalysis of the data in an effort to provide a more coherent explanation for the reported discrepancies.

## Methods

### NVVRS method

The NVVRS used an area probability approach from the military records of 8.2 million veterans. The sample included both men and women, enlisted and officers, and represented all branches of the military. They obtained data from a sample of male Vietnam Theater-Veterans (n = 1,200) and male Vietnam Era-Controls (n = 424), as well as from female veterans and both male and female civilians controls. Additional details of the sampling procedure are described elsewhere. [19] We report data only for the male Veterans in this report.

The diagnostic methods in the NVVRS were complex. Initially, a clinical subsample that disproportionately included PTSD subjects from both the Vietnam Theater-Veterans (n = 344) and Vietnam-Era Veterans (n = 96) was used to develop a series of logistic regression models that predicted a complex dichotomous variable based on multiple measures of PTSD. Subjects for the clinical subsample were selected to be within reasonable commuting distance of 28 specific Standard Metropolitan Statistical Areas (i.e., no farther than 75 miles from any of the 28 geographic areas). The clinical subsample included "apparent" PTSD cases and apparent non-cases. The apparent non-cases were selected to maximize the likelihood of detecting false negatives by over-sampling individuals with high scores on a combat exposure index and/or reporting nonspecific psychological distress.

The dichotomous outcome used for the clinical subsample logistic regression analyses was a composite PTSD diagnosis that comprised the MISS-PTSD[60], the Keane MMPI-PTSD sub-scale [65], and a semi-structured diagnostic interview based on FORM NP-V of the Structured Clinical Interview for DSM-III-R (SCID) [50,51,83]. Rules for interpreting and combining results from the three different instruments have been published previously. [82] The predictors for the logistic regression equations included the total score from the MISS-PTSD and the total number of PTSD symptoms reported from a structured diagnostic PTSD interview developed in the style similar to the Diagnostic Interview Schedule (D-PTSD). [53,56,84,85] The results of the logistic regression models from the clinical subsample were subsequently used to extrapolated prevalence estimates for PTSD for the entire NVVRS sample who were all administered the MISS-PTSD and the D-PTSD.

### VES methods

For the VES, 48,513 records were randomly sampled from an initial population of 4.9 million U.S. Army records. Records were selected for the study based on the following six criteria: 1) male veteran, 2) military occupational specialty (MOS) other than duty soldier or trainee, 3) single term of enlistment, 4) minimum of 16 weeks of active service time, 5) pay grade E-1 to E-5 at discharge, and 6) entered the military for the first time between January 1, 1965 and December 31, 1971. Only veterans who served in Vietnam at some time during their enlistment were included in the Vietnam Theater sample. To be included in the Vietnam-Era Control group, the veteran must have served at least one tour of duty in Germany, Korea, or the United States during the same time period, and never have served in the Army in Vietnam. The sample used to estimate PTSD prevalence rates in the VES was comprised of 4,462 veterans who voluntarily participated in an intensive one-week medical and psychological evaluation in 1985 and 1986. The sample was randomly drawn from a larger random sample of 15,288 veterans who participated in the telephone interview component of the VES. Both of these samples, as well as characteristics of non-respondents, are described in detail elsewhere. [21,86,87]

All subjects were administered a modified version of the Diagnostic Interview Schedule III-A (DIS-III-A), which included a PTSD module. Details of the DIS-III-A modifications have been documented previously. [87] In articles published by the CDC, [21] PTSD prevalence estimates were based solely on whether or not veterans met the diagnostic criteria for combat-related PTSD using the modified DIS-III-A. The MMPI was administered to all veterans and would have allowed for the scoring of the MMPI-PTSD sub-scale, but these results were not incorporated into published findings.

### Comparison of PTSD prevalence estimates for the VES and NVVRS

The primary comparisons for the two studies focused on the DSM-III-R criteria for combat-related PTSD. We also included alternative definitions of combat-related PTSD using criteria from both DSM-III and DSM-III-R. The DSM-III-R Manual [88] requires at least 6 symptoms for PTSD: one B-Criteria symptom, three C-Criteria symptoms, and two symptoms the D-Criteria symptoms. There are a total of 17 symptoms or probes that comprise the criteria: four B-Criteria symptoms, seven C-Criteria symptoms, and six D-Criteria symptoms.

Modified versions of the PTSD module from the Diagnostic Interview Schedule were the only instruments that were administered to all subjects in both studies. For the NVVRS, the D-PTSD module included 21 symptom probes, while the VES DIS-III-A PTSD module included only 9 symptom probes. Therefore, the NVVRS sampled more than the standard 17 DSM-III-R symptoms associated with PTSD while the VES sampled fewer probes.

The MMPI-PTSD was administered to all subjects in the VES and to the clinical sub-sample of the NVVRS. The MMPI-PTSD scale has been used with a number of different cutting-scores based on the types of cases who were important to identify and to differentiate. For example, low cutting-scores of 13 and 14.5 have been used in studies where investigators wanted to minimize the identification of false-negatives while a cutting-score of 30 has been used in studies where the investigators wanted to minimize the identification of false positives. [65,70,71] The MISS-PTSD was administered only to subjects in the NVVRS. A similarly sensitive cutting-score of 89 has been used in studies where investigators wanted to minimize the identification of false-negatives while a specific threshold of 107 has been used when attempting to minimize the identification of false positives. [19,60]

For the purposes of comparisons across the two studies, we constructed several alternative composite diagnoses. For the NVVRS, the D-PTSD and the MISS-PTSD were administered to all subjects in the total sample, and therefore these instruments were used to develop a composite diagnosis for combat-related-PTSD for the NVVRS sample. For the composite diagnosis, a score of 94 was used for the MISS-PTSD. Previous research has suggested that a score of 94 provides the most reliable estimate of the PTSD prevalence rate for Vietnam Veterans. [19] For the VES, the DIS-III-A PTSD and the MMPI-PTSD were administered to all subjects in the sample; thus these instruments were used to develop a multiple indicator diagnosis of combat-related-PTSD for the VES sample. For the composite diagnosis, a cutting-score of 26 was used for the MMPI-PTSD scale. The composite diagnosis was defined as a variable that ranged from zero to two and was estimated by summing the dichotomously coded variables from the two instruments used to estimate the prevalences. The composite score was then recoded into a "narrow" PTSD definition and a "broad" PTSD definition. For example, for the NVVRS, the D-PTSD and the MISS-PTSD were the two instruments used to provide the final diagnosis for PTSD. A person who was positively diagnosed with both the D-PTSD and the MISS-PTSD instruments would have been classified as a hit for both the narrow PTSD definition and the broad PTSD definition. A person who was diagnosed positively with just one of the two instruments would have been classified as a hit for only the broad PTSD definition. The results for the narrow definition are over-weighted toward the prevalence from the instrument with the lowest prevalence, while the results for the broad definition are overweighted toward the prevalence of the instrument with the highest prevalence. We were unable to make sensitivity and specificity estimates for either study because we did not have a gold standard measure. We were also unable to make direct comparisons of overlapping items from the two studies because many of the items from the DSM-III criteria were split into several different items in the DSM-III-R criteria.

Based on feedback from one of our reviewers, we carried out an analysis using the NVVRS sample comparing subjects who were "VES-Like" to subjects who were "Non-VES-Like". A similar analysis has been carried out previously on one of the outcome measures.[82]

## Results

### NVVRS

#### NVVRS total sample

The weighted prevalence estimates for DSM-III-R PTSD symptoms occurring currently (i.e. symptoms occurring within the last month), using the D-PTSD scale from the NVVRS, are presented in Table 1. For Criterion B symptoms, using DSM-III-R rules, the current prevalence estimates for the male Vietnam Theater-Veterans ranged from 1.8% for "flashbacks" to 3.4% for "disturbing memories". For the male Vietnam-Era Controls, the Criterion B symptom current prevalences ranged from 0.3% for "sudden anxious feeling" to 1.8% for "disturbing memories". For the criterion C symptoms, the highest current prevalence rate was "feeling cut off from people" for both groups of veterans. Finally, for Criterion D symptoms, the highest current prevalence estimate for the Theater-Veterans was "difficulty falling asleep" (5.9%). For the Era-Controls, the highest current prevalence was for "minor events making you angry" (2.6%).

**Table 1:** PTSD Items Administered Using D-PTSD Scale in NVVRS

**DSM-III-R Symptoms Occurring in Last 30 Days** **Criteria**		**Male Vietnam Theater Veterans**			**Male Vietnam Era Controls**		
		**N**	**Prev (%)**	**95% CI**	**N**	**Prev (%)**	**95% CI**
**B**	Unpleasant memories	1183	2.7	1.7–3.8	410	1.1	0.0–2.3
	Disturbing memories	1182	3.4	2.1–4.6	406	1.8	0.0–3.5
	Felt as if event recurring	1181	3.2	1.9–4.5	406	1.0	0.0–2.3
	Flashbacks	1179	1.8	0.9–2.7	404	0.5	0.0–1.3
	Suddenly feeling anxious/fearful/panicky	1187	3.3	2.0–4.7	407	0.3	0.0–0.5
**C**	Tried not to think about previous events	1179	3.1	1.9–4.4	408	0.7	0.0–1.5
	Avoided feelings about previous events	1185	2.8	1.6–4.0	403	0.3	0.0–1.0
	Avoided certain places or activities	1190	1.3	0.6–2.1	410	1.3	0.0–2.8
	Could not remember portions of traumatic event	1169	1.8	0.8–2.8	406	0.0	0.0–0.1
	Lost interest in activities	1188	2.8	1.6–4.0	410	1.2	0.0–2.5
	Stopped caring about activities	1190	2.0	1.0–3.0	411	1.4	0.0–2.9
	Felt cut off from people	1186	4.1	2.6–5.5	410	1.4	0.0–2.9
	Felt numb or empty inside	1191	2.3	1.2–3.4	411	1.2	0.0–2.4
	Could not feel things anymore	1189	2.5	1.4–3.6	408	0.6	0.0–1.6
**D**	Difficulty falling asleep	1182	5.9	4.1–7.6	410	2.2	0.5–3.9
	Felt as if you might get out of control	1185	1.5	0.7–2.3	406	1.0	0.0–2.2
	Minor events bothered you/made you angry	1189	3.1	1.9–4.3	411	2.6	0.1–4.7
	Trouble concentrating	1192	2.8	1.7–3.9	412	1.2	0.1–2.3
	Felt you had to stay on guard	1190	3.4	2.1–4.7	409	2.0	0.1–3.8
	Spells or panic attacks due to previous memory	1175	0.9	0.3–1.4	407	0.0	NA
	Unexpected noises startle you	1190	3.6	2.2–5.0	412	1.1	0.0–2.3

1. Confidence Intervals that were less than 0 were truncated to 0.

The weighted prevalence estimates for two different measures of PTSD used in the NVVRS sample are presented in Table 2. For the male Vietnam Theater-Veterans, PTSD prevalence estimates using the MISS-PTSD ranged from 6.0% to 20.9% depending on the cutting-score used. Current PTSD prevalence estimates using the D-PTSD were 3.3% for combat-related PTSD and 3.5% for PTSD due to any trauma. For lifetime PTSD prevalence estimates using the D-PTSD, the estimates were 7.9% and 8.9% for these same veterans.

**Table 2:** PTSD Scales Administered to Entire NVVRS Sample

**Scale**	**Criteria**	**Male Vietnam Theater Veterans**			**Male Vietnam Era Controls**		
		**N**	**Prev (%)**	**95% CI**	**N**	**Prev (%)**	**95% CI**
**Mississippi Scale**	**Cutting-Score ≥ 107**	1190	6.0	4.4–7.6	406	3.8	1.4–6.2
	**Cutting-Score ≥ 94**	1190	15.3	12.5–18.1	406	8.3	4.8–11.8
	**Cutting-Score ≥ 89**	1190	20.9	17.8–24.1	406	11.2	7.3–15.2
							
**One Month D-PTSD (DSM-III-R)**	**All-Cause PTSD**	1197	3.5	2.3–4.8	412	1.6	0.0–3.1
	**Combat-Related PTSD**	1197	3.3	2.1–4.6	412	0.0	0.0–0.3
							
**Lifetime D-PTSD (DSM-III-R)**	**All-Cause PTSD**	1197	8.9	6.9–10.8	412	4.6	2.2–7.1
	**Combat-Related PTSD**	1197	7.9	6.0–9.8	412	0.1	0.0–0.2

As expected, for the male Vietnam-Era Controls, current and lifetime PTSD prevalence estimates were substantially lower and ranged from 3.8% to 11.2% using the MISS-PTSD. Current PTSD prevalence estimates using the D-PTSD were 0.0% for combat-related PTSD and 1.6% for all-cause PTSD. Lifetime combat-related PTSD prevalence estimates using the D-PTSD were also quite low relative to the male Vietnam Theater-Veterans with prevalence of 0.1% for combat-related PTSD and to 4.6% for the all-cause PTSD.

#### Clinical subsample

Three PTSD instruments were administered to the NVVRS clinical sub-sample (i.e., MISS-PTSD, MMPI-PTSD, and the D-PTSD). The weighted prevalence estimates for the PTSD scales obtained from the clinical subsample are presented in Table 3. For the male Vietnam Theater-Veterans, the PTSD prevalence estimates ranged from 7.0% to 20.4% using the MISS-PTSD measure and ranged from 3.8 to 14.5 using the MMPI-PTSD instrument. For current PTSD prevalence rates using the D-PTSD, estimates were 3.3% and 3.4% for the male Vietnam Theater-Veterans. For lifetime PTSD prevalences using the D-PTSD, the estimates were 10.8% and 10.1% for the all-cause and combat related PTSD, respectively. Similarly for the Male Vietnam-Era Controls, the MISS-PTSD estimates ranged from 12.1% to 26.3% while the MMPI-PTSD ranged from 10.1% to 28.1%. For the D-PTSD, PTSD prevalence estimates ranged from 0.3% to 3.8% depending on the trauma and whether symptoms were experienced currently or previously.

**Table 3:** PTSD Scales Administered to NVVRS Clinical Subsample

**Scale**	**Criteria**	**Male Vietnam Theater Veterans**			**Male Vietnam Era Controls**		
		**N**	**Prev (%)**	**95% CI**	**N**	**Prev (%)**	**95% CI**
**Mississippi Scale**	**Cutting-Score ≥ 107**	259	7.0	3.8–10.2	55	12.1	1.0–23.2
	**Cutting-Score ≥ 94**	259	14.9	10.3–19.5	55	19.0	5.1–33.0
	**Cutting-Score ≥ 89**	259	20.4	14.7–26.1	55	26.3	16.0–36.6
							
**MMPI-Keane Scale**	**Cutting-Score ≥ 30**	232	3.8	1.0–6.6	50	10.1	0.0–23.2
	**Cutting-Score ≥ 26**	232	5.9	2.6–9.3	50	10.1	0.0–23.2
	**Cutting-Score ≥ 14**	232	14.5	9.9–19.2	50	28.1	11.8–44.3
							
**One Month D-PTSD (DSM-III-R)**	**All-Cause PTSD**	259	3.4	1.0–5.8	57	0.3	0.0–0.8
	**Combat-Related PTSD**	259	3.3	0.9–5.7	57	0.3	0.0–0.8
							
**Lifetime D-PTSD (DSM-III-R)**	**All-Cause PTSD**	259	10.8	6.3–15.2	57	3.8	0.0–10.8
	**Combat-Related PTSD**	259	10.1	5.8–14.5	57	0.3	0.0–10.8

1. Confidence Intervals that were less than 0 were truncated to 0.

### VES

The prevalence estimates for the individual items used for the DIS-III-A PTSD module are presented in Table 4. All data presented represent symptoms that occurred within the last month of the PTSD interview and many years after the war's end. For Criterion B symptoms, using the organizational structure of DSM-III-R, the current prevalence estimates ranged from 1.9% for "felt as if event recurring" to 7.6% for "recurrent thoughts or dreams" for the male Vietnam-Theater-Veterans. For the male Vietnam-Era Controls, the Criterion B symptom current prevalences did not exceed 0.1% for any of the three symptoms. For the Criterion C symptoms, the symptom with the highest current prevalence was "avoiding situations that remind the person of past experiences" for both groups. Finally, for Criterion D symptoms, the highest prevalence estimate for the Vietnam-Theater-Veterans was "feeling jumpy or easily startled "(10.6%). For the Era-Controls, the highest one month prevalence was the same item "feeling jumpy or easily startled" (0.3%).

**Table 4:** PTSD Items Administered Using DIS-III A Instrument in VES

**DSM-III-R Symptoms Occurring within Last Month** **Criteria**		***Male Vietnam Theater Veterans*** **(N = 2,483)**		***Male Vietnam Era Controls*** **(N = 1,976)**	
		**Prev (%)**	**95% CI**	**Prev (%)**	**95% CI**
**B**	**Recurrent thoughts or dreams**	7.6	6.6–8.6	0.1	0.0–0.3
	**Felt as if event recurring**	1.9	1.4–2.4	0.0	NA
	**Symptoms get worse in situations that remind**	3.9	3.1–4.7	0.1	0.0–0.3
**C**	**Lost ability to care/Lost interest in activities**	5.1	4.2–6.0	0.1	0.0–0.3
	**Avoids situations that remind**	7.9	6.8–9.0	0.2	0.0–0.4
	**Ashamed of being alive**	1.9	1.4–2.4	0.1	0.0–0.3
**D**	**Jumpy or easily startled**	10.6	9.4–11.8	0.3	0.0–0.6
	**Trouble Sleeping**	7.4	6.4–8.4	0.2	0.0–0.4
	**Forgetful or trouble concentrating**	5.2	4.3–6.1	0.1	0.0–0.3

The current prevalence estimates for the summary measures from the VES are presented in Table 5. For the Vietnam-Theater-Veterans, the MMPI-PTSD prevalence estimates based on various cutting scores ranged from 6.4% to 34.2%. For the male Vietnam Era-Controls, the MMPI-PTSD prevalence estimates ranged from 3.0% to 24.5%. For current PTSD prevalence estimates using the DIS-III-A PTSD instrument and DSM-III criteria, 2.4% of the male Vietnam-Theater-Veterans were positively diagnosed as cases of combat-related PTSD. When DSM-III-R criteria were used, the current PTSD prevalence estimate increased to 4.7%. There were no Vietnam-Era controls diagnosed with current combat-related PTSD using the DIS-III-A PTSD instrument.

**Table 5:** PTSD Scales Administered to VES Sample

**Scale Criteria**	**Criteria**	**Male Vietnam Theater Veterans**			**Male Vietnam Era Controls**		
		**N**	**Prev (%)**	**95% CI**	**N**	**Prev (%)**	**95% CI**
**MMPI-Keane Scale**	**Cutting-Score ≥ 30**	2,483	6.4	5.4–7.4	1,976	3.0	2.2–3.8
	**Cutting-Score ≥ 26**	2,483	10.0	8.8–11.2	1,976	4.8	3.9–5.7
	**Cutting-Score ≥ 14**	2,483	34.2	32.3–36.1	1,976	24.5	22.6–26.4
							
**One Month DIS-III-A PTSD**	**DSM-III Combat-Related PTSD**	2,483	2.4	1.8–3.0	1,976	0.0	0.0–0.1
	**DSM-III-R Combat-Related PTSD**	2,483	4.7	3.9–5.5	1,976	0.0	0.0–0.1
							
**Lifetime DIS-III-A PTSD**	**DSM-III Combat-Related PTSD**	2,483	14.7	13.3–16.1	1,976	0.6	0.3–0.9
	**DSM-III-R Combat-Related PTSD**	2,483	27.4	25.6–29.2	1,976	1.1	0.6–1.6

For lifetime combat-related PTSD prevalence estimates (i.e. now or ever in the lifetime), 14.7% of the male Vietnam Theater-Veterans were identified as cases using the DIS-III-A PTSD instrument and DSM-III criteria while 27.4% were characterized as cases using the DIS-III-A PTSD instrument and DSM-III-R criteria. A very small percentage of the Controls were identified as cases using the DIS-III-A PTSD instrument and lifetime DSM criteria for combat-related PTSD (0.6% for DSM-III and 1.1% for DSM-III-R). When DSM-III-R criteria were used, the PTSD prevalence estimates nearly doubled for both current and lifetime PTSD estimates.

### Comparisons of prevalence estimates: NVVRS vs. VES

Table 6 presents the composite estimates for combat-related PTSD from both samples. The composite diagnosis for the NVVRS used the cutting score of 94 for the MISS-PTSD while the composite diagnosis for the VES used a cutting score of 26 for the MMPI-PTSD. We focus on comparisons of DSM-III-R prevalence estimates as the estimates could be calculated from both studies. For the male Vietnam-Theater-Veterans, the narrow prevalence estimates for the NVVRS and VES were 2.9% and 2.5% respectively. The broad prevalence estimates were 15.8% and 12.2% respectively. For the male Vietnam-Era controls, a comparison of the prevalence rates across the two studies is less helpful given the focus on combat-related PTSD. For the narrow prevalence estimates, the NVVRS and VES both obtained estimates of 0.0% while for the broad estimates, the two studies yielded estimates of 8.3% and 4.8% respectively.

**Table 6:** Summary of Broad and Narrow PTSD Criteria for the NVVRS and VES Samples

**Scales**	**Criteria**	**Male Vietnam Theater Veterans**			**Male Vietnam Era Controls**		
		**N**	**Prev (%)**	**95% CI**	**N**	**Prev (%)**	**95% CI**
**NVVRS Current PTSD Diagnosis**	**Broad-DSM-III-R**^1^	1,188	15.8	12.9–18.6	406	8.4	4.9–11.9
	**Narrow-DSM-III-R**^1^	1,188	2.9	1.7–4.1	406	0.0	0.0–0.1
							
**VES Current PTSD Diagnosis**	**Broad-DSM-III**^2^	2,483	10.7	9.5–11.9	1,976	4.8	3.9–5.7
	**Narrow-DSM-III**^2^	2,483	1.7	1.2–2.2	1,976	0.0	NA
	**Broad-DSM-III-R**^1^	2,483	12.2	10.9–13.5	1,976	4.8	3.9–5.7
	**Narrow-DSM-III-R**^1^	2,483	2.5	1.9–3.1	1,976	0.0	NA

1. Based on DSM-III-R definition for the DIS instrument.

2. Based on DSM-III definition for the DIS instrument.

### Comparison of NVVRS "VES-Like" to "Non-VES-Like" subjects

Similar to the previous analyses carried out by the NVVRS research team [82], there were no statistically significant differences on any of the outcome measures comparing the "VES-Like" subjects to the "Non-VES-Like" subjects for either the male Vietnam-Theater-Veterans or the male Vietnam-Era-Veterans.

## Discussion

The results of this study provide additional insight into the sources of the disparate rates of PTSD reported in the VES and NVVRS, conducted by two independent groups of investigators. The use of a similar methodology to assess clinically relevant PTSD criteria and symptoms resulted in consistent and understandable results across the two studies for male Vietnam Theater-Veterans. Specifically, the prevalence estimates of combat-related PTSD for male Vietnam Theater-Veterans was 2.5% using the VES and 2.9% using the NVVRS with restrictive criteria. Using more sensitive thresholds, the estimates were also quite similar across the two studies; 12.2% using the VES and 15.8% using the NVVRS. The discrepancies in the originally reported prevalence rates [19,21] can be attributed to several factors:the NVVRS opted for more sensitive cutting-scores while the VES opted for more specific cutting-scores; the NVVRS used a six month prevalence estimate for current PTSD while the VES used a one month prevalence estimate for current PTSD and the VES used a single indicator for estimating prevalence whereas the NVVRS used multiple, fungible indicators.

In one study, the DIS-III-A PTSD measure was criticized as being an invalid measure of PTSD [82] and the poor validity of the instrument was put forward as the reason for the low prevalence estimates reported in the VES. Issues surrounding sensitivity and specificity often lead to the misinterpretation of similarities and differences in prevalence rates when comparing results across studies. [89-93] The results from our efforts at reconciliation suggest that the original VES study used very specific criteria for estimating the prevalence of PTSD (i.e. they only included symptoms that met criteria for each symptom and they used fewer symptom probes) while the NVVRS used very sensitive criteria for estimating the prevalence of PTSD (i.e. they included symptoms that were below criteria for each symptom and they included more symptom probes). Even though the DIS-III-A PTSD included fewer PTSD probes relative to the D-PTSD, we demonstrated that the DIS-III-A PTSD still retained enough items to provide useful data for estimating the prevalence of PTSD, especially when used in conjunction with another instrument such as the MMPI-PTSD.

Estimates of disease prevalence can vary dramatically depending on the time scale sampled (i.e. one month versus 6 month estimates). In establishing current PTSD, NVVRS inquired about PTSD symptoms recalled in the last 6 months while the VES inquired about symptoms in the last month. It should not be surprising that when we compared data for similar time frames following a severe trauma, the NVVRS and VES were more similar in terms of their prevalence estimates for PTSD.

Consistent and reliable measurement of complex psychological constructs such as PTSD is critical to advancing our understanding of both the etiology and treatment of psychological disorders. [72] The Institute of Medicine has recently been charged with reviewing the previous literature and recommending a reliable measure of PTSD for Gulf War veterans.[94] In general, single indicator models of psychological constructs are considered to be both less reliable and less valid measures compared to multiple indicator models. [72-74] Increasing the number of reliable probes used to measure psychological constructs results in an associated increase in the overall reliability of the instrument. For the NVVRS, the D-PTSD module included 21 symptom probes while for the VES study, the DIS-III-A PTSD module included only 9 symptom probes. Therefore, assuming equal reliability among the individual items, the NVVRS provided a more reliable estimate of combat-related PTSD prevalence relative to the VES. Finally, we agree with Kulka and colleagues [82] that the differences between the NVVRS and the VES were due to differences in instrumentation rather than due to differences in samples.

## Conclusion

The results of our study highlight the benefits of applying uniform criteria across studies when comparing results and help to resolve some of the previously reported discrepant results in the literature. We also highlight the implications of using different thresholds for estimating the prevalence of combat-related PTSD. We strongly suggest that future comparisons of combat-related PTSD prevalence estimates use available sensitivity and specificity data to adjust observed prevalence estimates in order to compare estimates of the true population prevalences, within and across wars to detect secular changes. Finally, and sadly, with the beginning of several new wars throughout the international community, future studies of combat-related PTSD should consider the use of similar PTSD instruments to the ones used in the VES and NVVRS to allow for direct comparisons of prevalence estimates and predisposing factors across wars.

## Competing interests

The author(s) declare that they have no competing interests.

## Authors' contributions

Dr. Thompson was responsible for writing the manuscript, data analyses, and interpretation of the results. Dr. Gottesman was responsible for acquiring data, interpretation of the results and revising and editing the manuscript. Dr. Zalewski was responsible for interpretation of the results and revising and editing the manuscript.

## Pre-publication history

The pre-publication history for this paper can be accessed here:


